# Body Condition Scores in Cats and Associations with Systolic Blood Pressure, Glucose Homeostasis, and Systemic Inflammation

**DOI:** 10.3390/vetsci11040151

**Published:** 2024-03-28

**Authors:** Rebeca Costa Vitor, Joana Thaisa Santos Oliveira, Adan William de Melo Navarro, Ana Carolina Ribeiro Lima, Gabriela Mota Sena de Oliveira, Alexandre Dias Munhoz, Anaiá da Paixão Sevá, Paula Elisa Brandão Guedes, Renata Santiago Alberto Carlos

**Affiliations:** Department of Agricultural and Environmental Sciences, State University of Santa Cruz (UESC), Ilhéus 45662-900, BA, Brazil; rebeca.scosta@hotmail.com (R.C.V.); joanathaisa@hotmail.com (J.T.S.O.); willnavarro2008@gmail.com (A.W.d.M.N.); acrlima99@gmail.com (A.C.R.L.); gabimpes@hotmail.com (G.M.S.d.O.); munhoz@uesc.br (A.D.M.); apseva@uesc.br (A.d.P.S.); pebguedes@uesc.br (P.E.B.G.)

**Keywords:** *diabetes mellitus*, hypertension, overweight

## Abstract

**Simple Summary:**

Obesity is a condition commonly observed in domestic cats, and it is associated with systemic diseases, such as *diabetes mellitus* and cardiovascular diseases. Therefore, this research aimed to evaluate the relationship between overweight and obesity in domestic cats with systolic blood pressure and circulating concentrations of glucose, fructosamine, and serum amyloid-A. The results indicated that obese cats presented high systolic blood pressure values, as well as high circulating concentrations of glucose. This demonstrates that obesity can result in comorbidities that reduce life expectancy and quality of life in cats.

**Abstract:**

Background: Feline obesity is the most common nutritional disease in cats. This study aimed to investigate the differences between systolic blood pressure (SBP) and circulating concentrations of glucose, fructosamine, and serum amyloid-A (SAA) in ideal-weight, overweight, and obese cats. Methods: The animals were divided into three groups: ideal-weight (BCS 5, N = 20), overweight (BCS 6, N = 20), and obese cats (BCS ≥ 7, N = 20). SBP, circulating concentrations of glucose, fructosamine, and SAA were evaluated. Results: The SBP values of the ideal-weight, overweight, and obese cats were 140.0 mmHg, 160.0 mmHg, and 160.0 mmHg, respectively. The blood glucose and fructosamine levels for the ideal, overweight, and obese cats were 104.0 mg/dL and 245.0 µmol/L, 123.0 mg/dL and 289.0 µmol/L, and 133.0 mg/dL and 275.0 µmol/L, respectively, for each group. The SAA values were <5 ug/mL in all the groups. The SBP values of the cats with ideal BCS were significantly lower compared to overweight (*p* = 0.019) and obese (*p* = 0.001) cats. The blood glucose values of obese cats were higher than those of ideal-weight cats (*p* = 0.029). There was no statistical difference between the groups for fructosamine and SAA. Conclusions: Obese cats had significantly higher SBP and blood glucose concentrations than ideal-weight cats, showing the effect of BSC on these parameters.

## 1. Introduction

Obesity has become the most common nutritional disease in cats, with multifactorial predisposing factors, including genetic, social, cultural, metabolic, endocrine, sexual, and environmental factors [1,2,3,4,5].

In these animals, diseases related to overweight and obesity include musculoskeletal disorders [6], hypertrophic cardiomyopathy [7,8], respiratory [6] and oral disease [9], lower urinary tract disease [10,11], neoplasms [9], and *diabetes mellitus* [1,9,12,13,14,15], the latter being more associated with obesity [1].

The body condition score (BCS) determination technique is frequently used to diagnose obesity in cats because it is easy to perform and low in cost [16]. It is a subjective and semi-quantitative method that evaluates the animal’s body condition through inspection and palpation. This method uses rating scales of five and nine points, the latter being described by Laflamme [17].

Glucose homeostasis depends on the activity of β cells, their endogenous production by the liver, and their use in peripheral tissues, especially muscles [18,19]. In obese cats, excess energy is stored in white adipose tissue in the form of triacylglycerol, which, in excess, causes changes in adipokine secretion and hypersecretion of pro-inflammatory adipokines [20]. These changes lead to insulin resistance, decreasing the insulin receptor sites and, consequently, the insulin response due to failure in glucose transport [19,21]. The onset of type II diabetes in obese cats is preceded and predicted by defects in insulin sensitivity, glucose tolerance, and glucose elimination [18]. Under normal conditions, glucose uptake by skeletal muscle is composed of several intracellular steps. However, when there is insulin resistance, metabolic dysfunction occurs, which results in impaired translocation of GLUT4-containing vesicles to the membrane, decreasing the ability of skeletal muscle and other tissues to absorb glucose into cells, leading to a hyperglycemic state [22].

Measuring fructosamine levels is used to differentiate feline *diabetes mellitus* from transient hyperglycemia caused by stress. This ketoamine results from glucose’s irreversible and enzymatic binding with serum proteins, mainly albumin. Its determination estimates the blood glucose concentration of the last two to three weeks [18,23].

White adipose tissue produces pro-inflammatory cytokines and acute-phase proteins, so it is believed that white adipose tissue is an important source of increased concentrations of these compounds in obese individuals. This indicates links between obesity, insulin resistance, and metabolic syndrome [19,21]. SAAs are acute-phase proteins expressed in the presence of inflammation in domestic animals. The serum concentration increases by 25% due to the stimulation of pro-inflammatory cytokines during the inflammation process. These proteins are considered quantitative disease biomarkers, highly sensitive to inflammation, but not so specific since the level of acute-phase proteins can also increase in non-inflammatory diseases [24,25]. The acute-phase proteins of the positive group respond to cytokines, and SAA is regulated by IL-1, IL-6, TNF-α, and glucocorticoids [26].

The pathogenesis that associates obesity with hypertension is not completely understood in felines [27]. However, it is known that excess weight in humans can induce hemodynamic dysfunctions, which result in a decrease in peripheral vascular resistance, generally inappropriate in obese individuals, leading to systemic arterial hypertension [28]. Thus, it can be inferred that a similar mechanism can also occur in cats [29]. Non-invasive diagnostic measurement methods are the most used in clinical practice [27,30,31], such as Doppler and oscillometric devices. These methods must follow a protocol in order to minimize external factors, such as the stress caused by handling the feline, influencing the values obtained. Therefore, the professional who conducts the exams must be experienced and qualified to do so, not only for the correct restraint of the animal but also for the selection of the most adequate material for the measurement. In addition, the value of the first measurement must be disregarded. These and other precautions to be followed in the protocol are crucial for the values obtained to be reliable [31].

Few studies have investigated the association between BCS in cats and *diabetes mellitus*, hypertension, and chronic low-grade systemic inflammation. This study aimed to investigate and compare the differences between SBP and circulating concentrations of glucose, fructosamine, and SAA in ideal-weight (BCS 4 and 5), overweight (BCS 6 and 7), and obese (BCS > 7) cats.

## 2. Materials and Methods

### 2.1. Ethical Considerations and Study Population

The study was approved by the Ethics Committee on the Use of Animals (CEUA) of State University of Santa Cruz (UESC), Ilhéus, Bahia, Brazil, under protocol No. 010/19. All procedures were conducted in accordance with the ARRIVE guidelines and followed the Cat-Friendly Practice guidelines. The animals were included in the study after formal consent of their owners.

The study included 60 cats regardless of breed and gender. As inclusion criteria, the cats must be castrated to eliminate the influence of sexual hormones [32], healthy on physical examination, and present complete blood count (CBC) and biochemical results (urea, creatinine, alkaline phosphatase, T4, and basal cortisol) within normal range for the species. Animals that showed stress leukogram were not included. Furthermore, they needed to fit into one of the three scores that will be described below, based on the scale described by Laflamme [17].

To define BCS, the cats were weighed and the same digital scale, previously tested with an object of known weight, was used for all animals in the study. The ranking proposed by LaFlamme [17] was used to determine BCS; this graduation classifies cats on a scale from 1 to 9, performed by the same evaluator, in which scores 4 and 5 were considered ideal-weight, 6 and 7 were overweight, and above 7 were considered obese cats. Subsequently, they were divided into three sample groups, with 20 cats each. Group 1 was composed of cats with ideal weight, group 2 with overweight cats, and group 3 with obese cats.

### 2.2. Measurement of Systolic Blood Pressure (SBP)

Clinical examination and blood collection of the cats were performed after 12 h of fasting at the owners’ residences. For SBP measurement, the cats were physically restrained by their owners, and one of the thoracic limbs was chosen to apply the alcohol and then the ultrasonic gel to the palmar metacarpal region. A veterinary ultrasonic Doppler system (DV 610V-Medmega, São Paulo, Brazil) was positioned in the trichotomized area, and the cuff used corresponded to approximately 30% of the circumference of the animal’s forelimb. Headphones (IG955-Samsung, Suwon, Republic of Korea) were attached to the Doppler and used directly in the evaluator’s ears so that the cats did not hear the noises emitted by the equipment, minimizing stress to the cats. The first two measurements were discarded, and then five consecutive measurements were recorded to obtain the AVERAGE result. Values < 140 mmHg were considered normal for the species [31].

### 2.3. Measurement of Blood Glucose, Fructosamine, and Serum Amyloid A

After measuring systolic blood pressure, 5 mL of blood was collected from the jugular vein. The blood was placed in tubes without EDTA for fructosamine and serum amyloid-A measurement (SAA). Glucose test was performed immediately after collection to avoid the consumption of glucose by red blood cells. A drop of blood was placed on the veterinary glucometer test strip (Gluco Calea-Wellion), and values up to 135 mg/dL were considered within the normal range for the species [33].

In the laboratory, after centrifugation (2500× *g* rpm, for 10 min) of the tube without EDTA, the serum was packed in aliquots and stored at −20 °C.

The fructosamine test was performed on a semiautomatic biochemical analyzer (BioPlus, Seoul, Republic of Korea) using a commercial fructosamine kit (Labtest Diagnóstica SA, Lagoa Santa, MG, Brazil) according to the manufacturer’s instructions. The fructosamine reference was 200–360 µmol/L [34]. The SAA test was performed with the V-check device, using the commercial Feline SAA kit (BioNote, Inc., Gyeonggi, Republic of Korea), according to the manufacturer’s instructions. The kit used performs quantitative measurement, which, according to the manufacturer, considers values below 5.00 ug/mL as normal.

### 2.4. Statistical Analysis

Since in cats age can influence blood pressure measurement [35] and age [18] and sex [32] increase the risk of developing *diabetes mellitus*, a multivariate analysis was performed, considering a significance level of 5%, for *p*-values and 95% of confidence intervals. To allocate cats into age groups, we used AAHA/AAFP Feline Life Stage Guidelines from 2021 [36]. All analyses and Boxplot graphics were performed with R software Version 3.5.0, using packages sjPlot and rstatix.

## 3. Results

The ideal-weight group had 70% (fourteen) females and 30% (six) males, whereas the overweight group had 40% (eight) females and 60% (twelve) males, and the obese group, 20% (four) females and 80% (sixteen) males. Related to animal age, the ideal-weight group varied between 1 and 7 years (median 3), the overweight between 1 and 9 (median 3), and obese between 2 and 13 (median 5). Related to body weight, the ideal-weight group varied between 2.5 and 4.6 kg (median 3.55 kg), the overweight between 4.4 and 5.8 kg (median 5.09 kg), and obese varied between 5.7 and 11.7 kg (median 6.95 kg). All the included cats were exclusively indoor and mixed breed.

**Figure 1:**
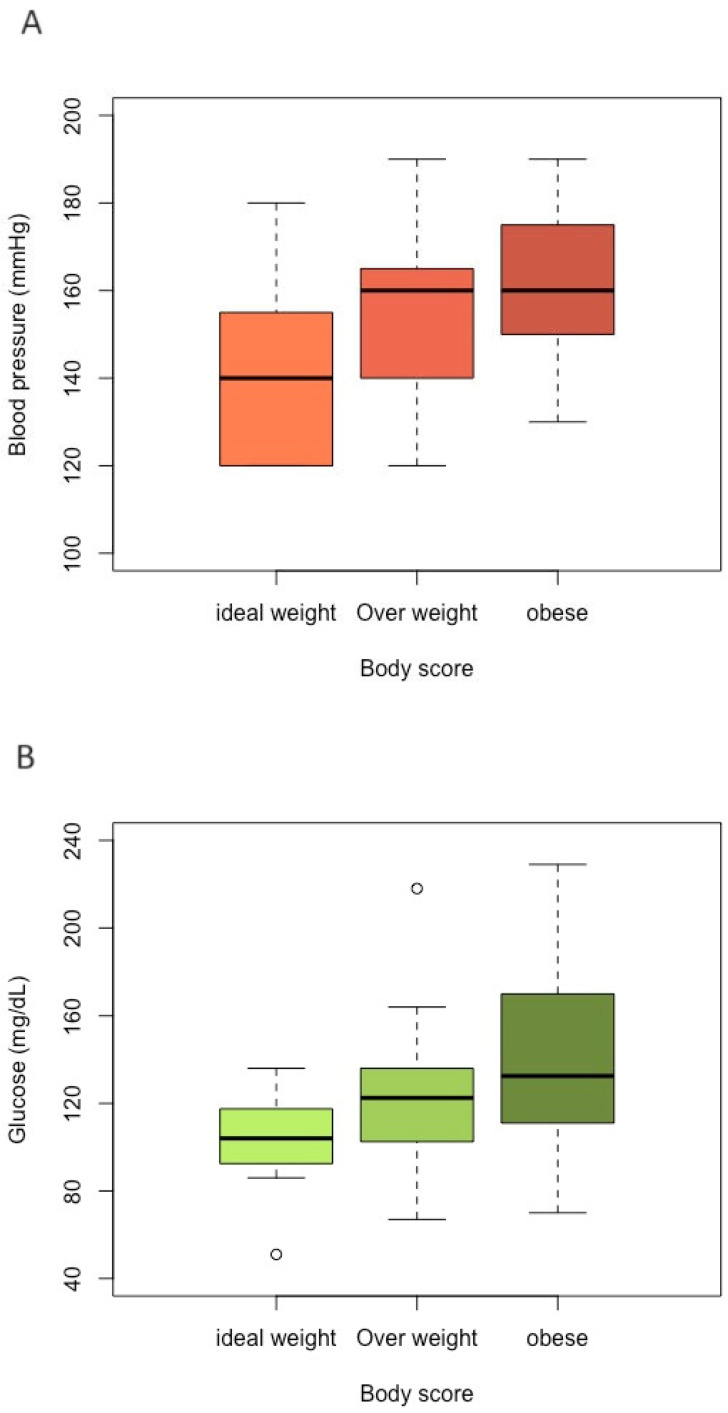

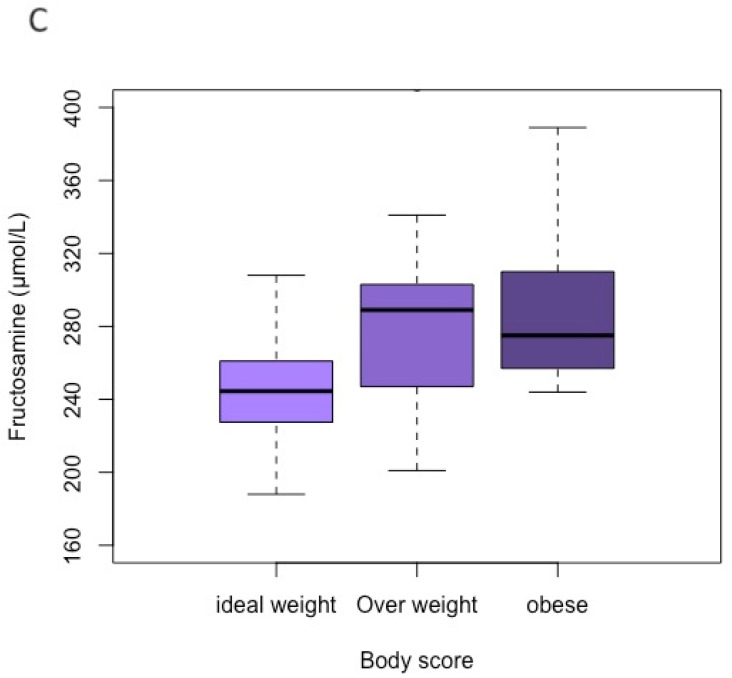
Boxplot for comparing each category of body group (ideal-weight, overweight, and obese) according to each variable value. (**A**) Blood pressure; (**B**) glucose; (**C**) fructosamine.

The median SBP values of ideal-weight, overweight, and obese cats were 140.0 mmHg (Interquartile Interval-IQR 120–153), 160.0 mmHg (IQR 140–163), and 160.0 mmHg (IQR 150–173), respectively (Figure 1A). For the blood pressure variable, gender and age did not show a statistically significant difference. Obese (*p* = 0.001) and overweight (*p* = 0.019) cats demonstrated statistical significance when compared with ideal-weight cats (Table 1).

For the variables glucosis and fructosamine, their values did not differ significantly according to gender and age group. For blood glucose, there was a difference only between obese and ideal-weight cats (*p* = 0.029). The median blood glucose levels of the ideal-weight, overweight, and obese groups were 104.0 mg/dL (IQR 93.3–115), 123.0 mg/dL (IQR 103–136), and 133.0 mg/dL (IQR 113–165), respectively (Figure 1B). For serum fructosamine concentration, there were no statistically significant differences among the BCS groups, and their median values of the ideal-weight, overweight, and obese groups were 245.0 µmol/L (IQR 228–259), 289.0 µmol/L (IQR 248–301), and 275.0 µmol/L (IQR 257–310), respectively (Figure 1C).

The SAA values of the ideal-weight, overweight, and obese cats were <5 ug/mL in all the groups. The SAA values did not differ significantly between the three cat groups. Of the 60 SAA tests, two cats of the same house had high values (183.1 ug/mL and 178.4 ug/mL), and, at the moment of blood collection, the CBC of these cats did not indicate any abnormalities. Two weeks later, both animals showed clinical signs compatible with feline rhinotracheitis. Amoxicillin plus potassium clavulanate were prescribed (20 mg/kg-BID for 15 days). One week after the end of treatment, the clinical signs disappeared, and a new evaluation of feline SAA was performed, and the result was within the normal range (<5 ug/mL).

## 4. Discussion

Overweight in felines, as well as in humans, has been increasing in worrying proportions [13]. For the diagnosis of obesity in the cats of the present study, the method used was an evaluation of BCS. This method was chosen for its practicality and low cost, without the need for anesthetic restraint. Despite being subjective, German [37] indicated that this method presents good repeatability and a good correlation with the percentage of fat obtained by dual-energy X-ray bone densitometry. This is the most accurate technique but is expensive and requires general anesthesia. However, the disadvantage of BCS is that it is indiscernible in animals with muscle loss and fat mass gain, so it is not recommended for general assessment of lean mass [4,37,38], which was not assessed in this study. So, this methodology was adequate for this evaluation.

Regarding systolic blood pressure, the statistical differences between the ideal-weight and overweight groups and the obese group indicate that excess weight predisposes to increased systolic blood pressure. The mechanism possibly involves hemodynamic changes caused by obesity, which involve humoral, hormonal, and inflammatory substances produced by adipose tissue [28,29]. It is worth mentioning that blood pressure values in cats should be interpreted with caution as it is known that the hospital environment, the presence of the veterinarian (due to the white coat effect), the physical examination, and the exact method of measuring SBP can lead to stress and, consequently, to a higher SBP [39]. In our study, the animals were evaluated in their homes, with their owners, and the evaluators using headphones to minimize the Doppler method’s discomfort also helped to reduce stress for the cats. As observers and researchers, we did not notice that overweight and obese animals were more stressed than ideal-weight cats.

Obesity in cats is a risk factor for *diabetes mellitus* [1]. Regarding blood glucose, the increase observed in the group of obese cats reveals the relationship between BCS and glycemia, as also observed by Billa et al., 2023 [40] in the research they carried out with groups of cats that were of ideal weight, overweight, and obese. Increased blood glucose with increasing BCS can be explained by low-grade systemic inflammation caused by obesity, which results in insulin resistance, due to the reduction in its receptor sites [38]. Since we found no correlation between SAA and obesity, it is likely that the increase in other inflammatory adipokines, such as TNF-α, which can promote insulin resistance at various levels, both centrally in the hypothalamus and within the adipocyte, is involved [41]. On the other hand, researchers revealed that TNF-α leads to increased production of acute-phase proteins, like SAA [42]. As weight gain along with inflammatory agents occurs gradually, a tendency towards insulin resistance follows this pattern [39,43].

As the group of obese cats had high blood glucose levels, it would be expected that the animals in the obese group would have high concentrations of fructosamine as it is a glycated serum protein that demonstrates long-term blood glucose concentration [44]. Although there was no statistical difference between the groups in the multivariate analysis, it was noted that the fructosamine levels had higher medians in accordance with the increase in BCS. Clinically, this increase in fructosamine levels observed in the groups of cats above the ideal weight, even though they remain within the normal values for the species, may suggest that, in the future, overweight or obese cats tend to develop *diabetes mellitus*. In this sense, according to Pérez-Lópes et al., 2020 [45], fructosamine values can be used as a tool for diagnosing pre-diabetes in cats. The results obtained point to the need to monitor obese cats.

It is worth mentioning that a limitation of the study was the difficulty in balancing the number of male and female cats in each group, as well as the ages in each group.

## 5. Conclusions

Obese cats had significantly higher SBP and blood glucose concentrations than ideal-weight cats. This change can result in comorbidities that can favor a reduction in life expectancy in overweight and obese cats. Under the conditions of this study, based on the measurement of SAA, systemic inflammation was not detected in either obese or overweight cats when compared to those with ideal weight.

## Figures and Tables

**Table 1:** Confidence interval (CI) estimates and *p*-values of multivariate analyses for comparison of glucosis, fructosamine, and blood pressure between sex, body condition score, and etary rate.

		Glucosis			Fructosamine			Blood Pressure		
Variable	Predictor	Estimates	CI	*p*-Value	Estimates	CI	*p*-Value	Estimates	CI	*p*-Value
Intercep of model		111.99	85.34–138.65	**<0.001**	258.91	218.04–299.78	**<0.001**	144.73	129.98–159.48	**<0.001**
Sex	Male	7.56	−12.40–27.52	0.458	16.93	−13.67–47.54	0.278	-		
	Female	Ref			Ref			-		
Body score	Obese	26.86	2.72–51.01	**0.029**	18.15	−18.87–55.17	0.337	19.22	7.44–30.99	**0.001**
	Overweight	13.13	−9.59–35.86	0.257	22.35	−12.49–57.19	0.209	14.16	2.33–25.98	**0.019**
	Ideal weight	Ref			Ref			Ref		
Etary rate	Senior	−14.12	−84.28–56.03	0.693	36.74	−70.83–144.31	0.503	−8.89	−47.55–29.77	0.652
	Mature adult	Ref			Ref			Ref		
	Young adult	−9.54	−34.02–14.95	0.445	−16.41	−53.95–21.13	0.392	−3.92	−16.89–9.04	0.553
	R^2^	0.162			0.115			0.19		

Legend. In bold are the significant *p*-values.

## Data Availability

No new data were created or analyzed in this study. Data sharing is not applicable to this article.
